# Supplementary material for: Exploring Ghanaian nurses knowledge and application of bio-ethical principles in postoperative pain management
Source: PLoS One. 2022 Oct 19;17(10):e0276422. doi: 10.1371/journal.pone.0276422 (PMC9581380; doi:10.1371/journal.pone.0276422)
Supplement: Attachment — Submitted filename: PONE-D-21-23036_reviewer.pdf [file pone.0276422.s001.pdf]

PLOS ONE

Exploring Ghanaian Nurses knowledge and application of bio-ethical principles in postoperative pain management.

--Manuscript Draft--

**Manuscript Number:**	PONE-D-21-23036
**Article Type:**	Research Article
**Full Title:**	Exploring Ghanaian Nurses knowledge and application of bio-ethical principles in postoperative pain management.
**Short Title:**	Knowledge and application of bio-ethical principles in surgical pain management
**Corresponding Author:**	Moses Banoya Tia<br>Methodist University College Ghana<br>Wenchi, GHANA
**Keywords:**	postoperative pain; bio-ethical; principles; Ghana; nurses
**Abstract:**	Managing postoperative pain require good understanding of the bio-ethical principles in order to preserve patients' rights. It is important that health care professionals understand that patients in pain have the right to satisfactory management. Good insight on ethical principles and how they relate to pain management places the nurse on a better pedestal to manage postoperative pain effectively. However, there is scanty literature on the level of Ghanaian nurses' knowledge and application of bio-ethical principles in postoperative pain management. The study aimed at exploring nurses' understanding and application of the bio-ethical principles in postoperative pain management.
**Order of Authors:**	Moses Banoya Tia
	Lydia Aziato
	Gladys Dzansi
**Additional Information:**	
**Question**	**Response**
**Financial Disclosure**<br><br>Enter a financial disclosure statement that describes the sources of funding for the work included in this submission. Review the submission guidelines for detailed requirements. View published research articles from *PLOS ONE* for specific examples.<br><br>This statement is required for submission and **will appear in the published article** if the submission is accepted. Please make sure it is accurate.	authors received no funding for this work

Powered by Editorial Manager® and ProduXion Manager® from Aries Systems Corporation

**Unfunded studies**<br>Enter: *The author(s) received no specific funding for this work.*<br><br>**Funded studies**<br>Enter a statement with the following details:<br>• Initials of the authors who received each award<br>• Grant numbers awarded to each author<br>• The full name of each funder<br>• URL of each funder website<br>• Did the sponsors or funders play any role in the study design, data collection and analysis, decision to publish, or preparation of the manuscript?<br>• **NO** - Include this sentence at the end of your statement: *The funders had no role in study design, data collection and analysis, decision to publish, or preparation of the manuscript.*<br>• **YES** - Specify the role(s) played.<br><br>* typeset	
**Competing Interests**<br><br>Use the instructions below to enter a competing interest statement for this submission. On behalf of all authors, disclose any competing interests that could be perceived to bias this work—acknowledging all financial support and any other relevant financial or non-financial competing interests.<br><br>This statement is required for submission and **will appear in the published article** if the submission is accepted. Please make sure it is accurate and that any funding sources listed in your Funding Information later in the submission form are also declared in your Financial Disclosure statement.<br><br>View published research articles from *PLOS ONE* for specific examples.	The authors have declared that no competing interests exist

**NO authors have competing interests**<br><br>Enter: *The authors have declared that no competing interests exist.*<br><br>**Authors with competing interests**<br><br>Enter competing interest details beginning with this statement:<br><br>*I have read the journal's policy and the authors of this manuscript have the following competing interests: [insert competing interests here]*<br><br>* typeset	
**Ethics Statement**<br><br>Enter an ethics statement for this submission. This statement is required if the study involved:<br><br>• Human participants<br>• Human specimens or tissue<br>• Vertebrate animals or cephalopods<br>• Vertebrate embryos or tissues<br>• Field research<br><br>Write "N/A" if the submission does not require an ethics statement.<br><br>General guidance is provided below. Consult the submission guidelines for detailed instructions. **Make sure that all information entered here is included in the Methods section of the manuscript.**	Noguchi Memorial Institute for Medical Research Institutional Review Board (CPN 016/17-18).<br>Ghana Health Service Ethics Review Committee (GHS-ERC: 012/10/17)<br>written consent were given.

Powered by Editorial Manager® and ProduXion Manager® from Aries Systems Corporation

**Format for specific study types**<br><br>**Human Subject Research (involving human participants and/or tissue)**<br>• Give the name of the institutional review board or ethics committee that approved the study<br>• Include the approval number and/or a statement indicating approval of this research<br>• Indicate the form of consent obtained (written/oral) or the reason that consent was not obtained (e.g. the data were analyzed anonymously)<br><br>**Animal Research (involving vertebrate animals, embryos or tissues)**<br>• Provide the name of the Institutional Animal Care and Use Committee (IACUC) or other relevant ethics board that reviewed the study protocol, and indicate whether they approved this research or granted a formal waiver of ethical approval<br>• Include an approval number if one was obtained<br>• If the study involved *non-human primates*, add *additional details* about animal welfare and steps taken to ameliorate suffering<br>• If anesthesia, euthanasia, or any kind of animal sacrifice is part of the study, include briefly which substances and/or methods were applied<br><br>**Field Research**<br><br>Include the following details if this study involves the collection of plant, animal, or other materials from a natural setting:<br>• Field permit number<br>• Name of the institution or relevant body that granted permission	
**Data Availability**<br><br>Authors are required to make all data underlying the findings described fully available, without restriction, and from the time of publication. PLOS allows rare exceptions to address legal and ethical concerns. See the PLOS Data Policy and FAQ for detailed information.	Yes - all data are fully available without restriction

Powered by Editorial Manager® and ProduXion Manager® from Aries Systems Corporation

A Data Availability Statement describing where the data can be found is required at submission. Your answers to this question constitute the Data Availability Statement and **will be published in the article**, if accepted.<br><br>**Important:** Stating 'data available on request from the author' is not sufficient. If your data are only available upon request, select 'No' for the first question and explain your exceptional situation in the text box.<br><br>Do the authors confirm that all data underlying the findings described in their manuscript are fully available without restriction?	
**Describe where the data may be found in full sentences. If you are copying our sample text, replace any instances of XXX with the appropriate details.**<br><br>• If the data are **held or will be held in a public repository**, include URLs, accession numbers or DOIs. If this information will only be available after acceptance, indicate this by ticking the box below. For example: *All XXX files are available from the XXX database (accession number(s) XXX, XXX.).*<br>• If the data are all contained **within the manuscript and/or Supporting Information files**, enter the following: *All relevant data are within the manuscript and its Supporting Information files.*<br>• If neither of these applies but you are able to provide **details of access elsewhere**, with or without limitations, please do so. For example:<br><br>*Data cannot be shared publicly because of [XXX]. Data are available from the XXX Institutional Data Access / Ethics Committee (contact via XXX) for researchers who meet the criteria for access to confidential data.*<br><br>*The data underlying the results presented in the study are available from (include the name of the third party*	All relevant data are within the manuscript

and contact information or URL).<br>• This text is appropriate if the data are owned by a third party and authors do not have permission to share the data.<br><br>* typeset	
Additional data availability information:	

Powered by Editorial Manager® and ProduXion Manager® from Aries Systems Corporation

Manuscript
Click here to access/download;Manuscript;KNOWLEDGE AND APPLICATION OF BIOETHICAL PRINCIPLES.docx

Title: Exploring Ghanaian Nurses knowledge and application of bio-ethical principles in postoperative pain management.

Authors' list:

Moses Banoya Tia[1]

Email: mbtia@st.ug.edu.gh

Lydia Aziato[2]

Email: aziatol@yahoo.com

Gladys Dzansi[2]

Email: gladysdzansi@gmail.com

Authors' affiliation

Methodist University College Ghana[1]

University of Ghana, Ghana [2]

// Abstract

Managing postoperative pain require good understanding of the bio-ethical principles in order to preserve patients' rights. It is important that health care professionals understand that patients in pain have the right to satisfactory management. Good insight on ethical principles and how they relate to pain management places the nurse on a better pedestal to manage postoperative pain effectively. However, there is scanty literature on the level of Ghanaian nurses' knowledge and application of bio-ethical principles in postoperative pain management. The study aimed at exploring nurses' understanding and application of the bio-ethical principles in postoperative pain management.

The study employed qualitative exploratory descriptive design. Purposive sampling technique was used to recruit participants from the surgical wards. Semi-structured interview guide was designed for data collection. Data saturation was reached at the fourteenth participant. Thematic analysis method was used and themes emerged inductively.

Four main themes identified through inductive content analysis of data were: generosity, empowerment to enhance autonomy, and fair treatment of patients. Findings showed that nurses had some appreciable level of knowledge of the fundamental principles related to ethics and applied them in postoperative pain management. Nurses knew their duties in advocating for patients. Patients rights to refuse treatment was also appreciated by some nurses. Nurses also demonstrated humanity by helping patients financially to settle hospital debts which explicitly shows the empathetic characteristics of nurses.

The study concluded that nurses are knowledgeable in bioethical principles underpinning post operative pain management and also applied these principles when caring for surgical patients. Thus, nurses need motivation as they suppport patients in various ways, such as financial assistance.

Keywords: postoperative pain; bio-ethical principles; Ghana.

Introduction

Ethics is important in the nursing profession. Ethics guide nurses in their practice on a daily basis to distinguish between right and wrong when the correct path is unclear in professional care environment (1). Nurses are expected to work in an ethical manner in order to meet the individual needs of patients and enhance satisfaction. Nurses who are knowledgeable about ethical theories may be better prepared for ethical nursing practice as they will have thorough knowledge of the most appropriate ethical approaches in a given situation (2). Ethics also enable nurses with the power to influence the day-to-day decisions that are made regarding patients care (3).

The underlying universal principles of ethics important for nursing practice are based on the obligation to do good (beneficence); do no harm (nonmaleficence); providing equal and fair treatment for all persons (justice); defending individual determination (autonomy) (4,5). These ethical principles provide a framework for the analysis and resolution of moral problems encountered in the delivery of healthcare. They form the basis for all codes of ethics and also the foundation of considerations for all professional groups in healthcare, including the nursing profession (6). Thus, in making decision to care for patients with postoperative pain, these principles influence nurses to achieve the objectives of care.

Beneficence is an important ethical principle that underpins nursing care and is basically the duty to do good to patients while considering their desires (7). Beneficence and non-maleficence are closely related. Beneficence is the principle of doing good (7–9). Beneficence involve the active promotion of benevolent acts such as goodness, kindness, charity (10). It may also include the injunction from inflicting harm. Nurses are obliged to implement actions that benefit patients and their support persons. Intentional harm is never acceptable in nursing. However, placing a patient at risk of harm has different facets. Unintentional harm is when the risk could not have been anticipated. Nonmaleficence is avoiding the causation of harm (7–9). It was argued that providing comfort can be deemed as an essential component of beneficence (11). Comfort needs of patients should be viewed as beneficence since it involves providing measures to relief pain. A phenomenological study conducted to explore nurses' and patients' views of comfort needs showed that comfort is an indispensable human need whether in illness or in good health (12). Comfort was seen as a state of being free from suffering and being in a calm environment. This therefore presupposes that nurses' efforts to provide comfort for their patients can be viewed as benevolent acts and professional duties.

Beneficent actions of nurses could be thwarted when they are confronted with workload and less staffing since time may not be sufficient to perform the core duties of care. A study to evaluate the impact of understaffed nursing shifts on patient outcomes in Australia showed that patient care quality dwindled with associated effects such as surgical wound infections, urinary tract infections, deep vein thrombosis, pressure injuries, and pneumonia (13) suggesting that such patients' comfort would be compromised.

Autonomy refers to the right to make one's own decisions. In contemporary discourse it has broad meanings, including individual rights (14,15), privacy, and choice (16). Autonomy entails the ability to make a choice free from external constraints. Patient autonomy is viewed as giving chance to make choices from options that have been made known to them regarding their own treatment. Autonomy involves allowing the patients to decide on what health interventions they wish to be provided for them (17).

Nurses who adhere to the principle of autonomy understand that each patient is unique and has the right to behave differently and make choices and set goals concerning treatment. Honouring the principle of autonomy require that the nurse respect patients' decisions even if those decisions are not in the best interest of the patient (18). The nurse has to also be considerate. Autonomy is violated in nursing care when a patient complain of pain is considered as exaggeration or demand for attention and thus, is not given the attention deserved. Also, when decision making processes are weak, patient autonomy is easily offended (19).

pain is described as subjective phenomenon and is better perceived, assessed and treated just as the individual in pain explains it (20). This explanation could be better understood in the context of patient autonomy. A study also elucidated that the subjective nature of pain is best treated when nurses understand and view pain as the patients says it is (21). By this, patients will feel comfortable since their views are accepted. This could depict that nurses acknowledge patients' autonomy regarding their expression of pain.

An important aspect of enhancing autonomy is through patient education. It was found in a study that patients empowerment is best achieved through education so that they are in a position to make informed choices (22). The study further noted that patients became knowledgeable about their conditions and were better able to carefully make informed decisions with education. These findings buttress the role of empowerment in providing patients with the opportunity to have control of their health. By this, patients may feel they have roles in ensuring their own good health and thus, participate actively towards recovery. Other studies, however, showed that patients preferred shared decision making rather than being completely autonomous(23,24).

Justice is often referred to as fairness and equality (18). Nurses face issues of justice every day as they organize care for their clients. They must decide how much time they have to spend with each client, taking patient needs into consideration, and then fairly distributing the resources accordingly. Justice as an ethical principle in nursing deals with fair treatment of patients and ensuring that the rights of individual patients are upheld (25). Alzheimer Europe describes justice as the moral obligation to act on the basis of fair adjudication between competing claims (8). It is further linked to entitlement, and equality. Justice is also seen as impartiality and objectivity towards patients irrespective of their social status, race, colour (26,27).

Advocacy can largely enhance justice. Advocacy roles played by nurses is considered as an integral element in ensuring safe practice in health care settings; advocacy for patients help in mitigating risks arising from sudden changes in patients' conditions and possible harm that may occur from misjudgement in treatment by other professionals (28). Furthermore, protecting patients' rights is noted to be an aspect of advocating in the interest of patients (29). A cross-sectional study in Uganda on nurses' knowledge of ethics showed that only 18 (15.8%) of the nurses and midwives obtained a score ≥50% in the ethics knowledge assessment test. Nurses who had diploma or higher education were less likely to score below 50% in the knowledge test as compared with nurses who had obtained only certificates (30). It was further noted that nurses with higher education scored higher in ethics knowledge test (30). Studies have also shown that nurses with long years of service, higher educational attainment and belonging to a professional nursing association exhibited high professionalism attributes such as autonomy, accountability, advocacy, collaboration and collegiality (31).

There is an established positive correlation between professional autonomy and higher education on attitude towards caring for dying patients (32) which presupposes that higher carrier development and years of experience are most likely to positively influence professional ethical standards in nursing care including ethical decision making for postoperative pain management. Studies also revealed that nurse practitioners who had knowledge of professional ethics such as autonomy were confident in healthcare delivery decisions which improved their quality of work (33). This presupposes that as professional nurses know ethics relating to pain management, they will give satisfactory care.

The authors extrapolated from previous studies on ethical principles since there is absolutely no single study on nurses' knowledge of bio-ethical principles regarding pain management. It is against this background that there is need to explore how nurses view the ethical principles and apply in nursing care of surgical pain.

Methods

Design

In order to explore nurses' knowledge of the ethical principles and their application to postoperative pain management, the researchers made use of qualitative exploratory descriptive design. This design was used in order to enable the researchers allow flexibility for participants' responses, and also owing to the fact that little is known about the topic under study as suggested by (34). This design allows for use of open-ended questions for exploring of a process, a variable or a concept that is not yet fully known or understood (35). Exploratory descriptive design is amenable to health environments research because it provides factual responses to questions about how people feel, about a space, and what reasons they have for using features of a space (36). The design therefore, enabled the researchers to gain understanding of the nurses' level of knowledge and how they applied bio-ethical principles in pain management by providing flexible questions where respondents could express themselves in detail.

Setting

This study was conducted in a Regional hospital in Ghana. The facility serves a major referral centre for surgical conditions from the all other facilities under the region. It has a large surgery department which is divided into male and female wards. Patients who undergo various major and minor surgical interventions are received and managed in this department. Research participants were obtained from both male and female adult surgical wards of the hospital.

Population and sampling technique

The target population was registered general nurses with diploma in nursing. The sampling technique used to recruit participants into the study was purposive sampling. This sampling technique was suitable since it enabled the researchers with the opportunity to select respondents who were believed to have knowledge of postoperative pain management. This was because the researchers needed participants who could respond to the interview questions and were also willing to participate. Registered general nurses with a minimum of diploma certificates and one year minimum working experience were eligible to participate. Nurses on leave were excluded from the study. The researchers approached potential participants on duty

Total

and explained the purpose of the study to gain consent for interview. total of 14 participants were interviewed.

Data collection and process

The researchers designed a semi-structured interview guide for data collection process. Individual interviews were conducted on participants and responses were audio taped. The interview guide comprised of open-ended questions which allowed participants to express their views well.Some of the main interview questions asked were: “explain some of the bioethical principles,” “How do you make use of the bioethical principles when managing patients’ pain in the surgical ward?” Iterative questions were also posed in order to gain clarity on ambiguous response. In situations where there was need to gain depth of explanations, further probing were done on participants. Interviews were conducted in English language and transcribed verbatim. Also, field notes were taken in order to help confirm responses from participants. Fields notes included non-verbal cues and expressions. Data collection took place from January to February 2018. Each interview lasted for an average of 45 minutes. The first researcher carried out the data collection.

Data analysis

Transcribed data were read through severally by the first researcher in order to gain full understanding of text. Content analysis method was used in analysing transcribed data. In content analysis, important themes and patterns that emerge from participants are identified (37). It involves identifying and condensing meaning units of words or texts (38). The approach was used to identify emerging themes from the data. Codes were generated from data and themes were subsequently obtained inductively. Verbatim quotes were used to support claims made by the researchers. Data transcription, coding, and analysis were done by the first author while the second and third authors verified results and ensured that the participants views were accurately represented.

Rigor

According to (39–41) credibility establishes whether or not the findings of a research represent plausible information obtained from respondents’ original views in the data obtained. Member checking, the heart of credibility (42) was employed. Analysed and interpreted data were given to respective participants to evaluate the interpretations made by the researchers.

Transferability is described as the degree to which qualitative research findings easily transferred to other contexts with other participants (39). Transferability was ensured by selecting participants who were believed to be able to respond to the interview questions that would address the research objectives. Participants' privacy and anonymity were also guaranteed to enable them express their views without fear of being heard and identified by others. This approach enhanced honesty of the participants regarding information they were given in the interview process. These ensured dependability of participants' information. Also, data collection instrument dependability was guarranteed through a pre-testing of the interview guide. This was done in a different health facility that had similar characteristics as the facility the study was conducted.

Demographics

There were fourteen (14) paricipants including three (3) males and eleven (11) females. Seven (7) each of the participants were from male and female surgical wards. Participants' ages ranged from 24 to 40 years. Six (6) were 35 years and above, and the rest were between the ages of 24 and 30 years. Six (6) were married while eight (8) were singles. Five (5) participants had more than 10 years of work experience, and nine (9) had between one (1) and ten (10) years work experience. Nine participants had diploma in nursing and the rest had bachelors degrees in nursing. All participants were general nurses with diploma in general nursing as a minimum qualification.

Research ethical consideration

Ethical clearance was obtained from Noguchi Memorial Institute for Medical Research Institutional Review Board and Ghana Health Service Ethics Review Committee with clearance numbers CPN 016/17-18 and GHS-ERC: 012/10/17 respectively. Each participant signed written informed consent form. The forms emphasised confidentiality, anonymity, voluntariness and participant's right to withdraw from study at any time without consequences. Anonymity of participants was ensured by the use of codes, while confidentiality was maintained through invididual interviews in privacy according to participants' discretion.

Results

Three main themes emerged from the data analysis. These are generosity, empowerment to enhance autonomy and fair treatment of patients. Two sub-themes were obtained from

"generosity": financial support for patients, and kindness with patients. The theme "empowerment of enhance autonomy" also had the following sub-themes: patients' empowerment and nurses' empowerment. The third theme also had two sub-themes, namely, non-discrimination of patients advocating for patients. Verbatim quotations are used to back the subthemes in the presentation of the results.

Generosity

Financial support for patients

Some of the participants said that they rendered various forms of help to patients. They said it was part of their inherent moral duty. Some examples of help that participants mentioned were giving money to patients to buy medications: "*Sometimes we the nurses buy food for patients who canntot afford.*" **(FN7).** Other nurses indicated that they supported patients with money for food. One of the nurses said that hunger is sometimes a reason for their pain and thus, helped those in serious need: *"some of the patients may afford the analgesics but are hungry. You can't also give some of these drugs on empty stomach. We just sometimes look for money to get them food." (***MN4).**

Some patients who were unable to pay their bills during discharge were helped by nurse: "*…other times we help in other things after discharge, like money for them to settle some bills."* **(FN1).**

Nurses also extended help to patients who had surgical operations and did not have enough money for analgesics. One nurse narrated:

"for some of our patients, I sometimes add up my few coins to be able to get their analgesics from the pharmacy because you don't even see their relatives tell them that more money is needed for their patient's treatment." **(FN14).**

Kindness with patients

Participants also said that it is their duty to make patients comfortable by managing their pain. One participant said: *"...making the patient feel free of pain, that is to avert or alleviate the pain or reduce it as much as I can.*" (**FN7**).

Another participant described her duties to include actions that bring about good results and recovery of patients. An example mentioned was administering pain medication before dressing surgical wounds:

> "*it is simply ensuring that your nursing actions are intended to produce good results so that the patient can recover fast…I will give pain killer maybe thirty minutes before I dress the wound.*" (**FN13**)

Others stated that they go ahead and administer pain medications whenever the patients complain of pain. Nurses said they would not withhold analgesics from patients in pain:

"I am always kind to the patients with their analgesics once I hear of any complain of pain from my patients." (**MN4)**

"*I have no reason to keep analgesics in the name of addiction when my patients are showing signs of pain. I give them PRN with the slightest complain to avoid worsening pain" (***MN 9).**

Participants also said that they tried to be generous with patients' prescribed medications:

"We try to ensure that we are generous with medications that are prescribed…without discriminating." **(FN7).**

some of the participants also indicated that spending more time outside of their schedules with patients especially in cases of staff shortage. A participants said: *"sometimes we just stay longer for the sake of the patients, just to give give extra helping hand when we are having shortage. Sometimes for a whole extra shift outside my schedule." (***FN13)**

Empowerment to enhance autonomy

Patients' empowerment

Participants said they were always giving patients opportunities in decision making: *"…the patient has the opportunity to make suggestions about himself regarding their pain treatment."* **(MN4)**

Some respondents also noted that making treatment options available was integral empowerment strategies for patients: *"The patient is supposed to be aware of all the things that may happen to him so that he can choose whatever he wishes."* (**MN9)**

Other participants said that educating patients on their conditions and the available treatment would make them get involved in their treatment: *"…you should explain to the patient what he should know so that he can be involved in whatever you are doing to him."* **(MN4).**

Seven of participants considered that the subjective nature of pain was better managed by allowing patient make informed decisions. One of them narrated:

"The patient has the right to express his pain and request for medication because he alone can tell how severe the pain is. For me I think it is important we even allow or tell them to inform us when they are in pain. They are even helping us by telling us how they feel" **(FN14)**

Only one participant noted the need to document patient's refusal of procedures in their attempts to exercise autonomy:

"…if you are going to do something for the patient, if the patient does not want it, he doesn't want it. You cannot force the patient to take it. Yours is that you have to document it:" **(MN9)**.

Other participants mentioned the need to make senior staff aware of patient's wilful refusal of certain treatment:

"Like a patient can say I don't like this medication. You can't force him to take it… then you may let your in-charge know that you did your best but patient refused" **(FN1)**

Nurses' Empowerment

Some of the nurses said that they were able to make decisions on patients' care when doctors were not on the wards:

"When the doctors are not around sometimes you just know that if I do this it will work so sometimes you just do it. You just take your own independent decisions and it help the patients" **(FN7)**

Other participants were able to assess patients with acute pain using the numeric rating scale and made prescriptions of analgesics for patients:

"Sometimes we are able to assess and score patients pain, especially those with acute pain and write medication. We go to the pharmacy and explain and by seeing the pain score they serve us and we give to the patient and document." **(FN14)**

FN7 said that they were involved in decision making. She noted that making decisions with doctors concerning patients treatment was considered teamwork and the doctors recognised nurses' ability in decision making: *"Sometimes on rounds we make inputs and decisions with the doctors. They appreciate nursing is autonomous body…*" **(FN7)**

Fair treatment of patients

Non-discrimination of patients

A participant said that non-discriminatory attitude towards patients is achieved by showing respect and treating them equally:

"…I consider all patients as equal and so I respect and treat them without discrimination. They are given their medications as they deserve especially the pain medications" **(FN11)**

Some participants indicated that attending to patients should not be on the basis of patients' social class or race:

> *"…we treat them equally despite their colour, race, whatever…so far as you are working with the individual and as it says, you should treat all of them equally, so you will work on that"* **(FN13)**

Some also said that since pain is subjective, patients should be allowed to express their feelings about pain and not disciminate how they may want to put it . One of them stated: *"…and the patient has the right to express their pain. We know that pain is subjective and patients should be allowed to say how they feel."*(**FN2).**

A participant also said they do not withhold prescribed pain medications from patients since it could be seen as discrimination:

> *"…If the pain killers are there, you have to administer…otherwise, when others complain and you give, it will be said that you discriminate"* **(MN9)**.

Many participants said there should be fair treatment of patients, such as being attentive to patients' needs and being non-discriminatory:

> *"You should make sure you treat them equally. Don't say because this one's father is a minister should get that, and this one should get that… I think being fair sometimes could be giving them the kind of attention they need"* **(FN3).**

Others said nurses should not be selective when attending to patients:

> *"If you are not attending to them don't attend to them at all. If you are doing stuff, do it but if you attend to one call, if the next person calls, you are supposed to go or else they will start saying something."* (**FN10).**

Advocating for patients

Participants noted that nurses are involved in advocating for patients in situations where they may not have the ability to do so. One of them narrated:

> *"I remember we had a patient who came here with road traffic accident and for just some few hours of stay in the hospital, they billed him about seven hundred cedis (GHC 700) which he had nothing to pay. It was only paracetamol that we gave and the paracetamol too he bought so we said no to this injustice. We had to send the folder back ourselves and explained to them and the bill was corrected to forty-seven cedis (GHC 47.00) and he paid and left."* **(FN2).**

Some nurses alluded that they explained to patients during the preoperative period about their rights to request for pain medications: *"…and we also try to explain to the patients that they can make request for analgesics when they come back from theatre"* **FN13.**

Some of the younger staff also said that they had to convince their senior nurses on duty before they administered analgesics to patients who were considered to exagerate their pain. A nurse stated:

> *"our seniors who have worked for long sometimes tell us that this patient is faking his pain. But sometimes I take the pain to check the patient and persuade my senior on duty to allow me give at least paracetamol"* **Fn1.**

Discussion

Nurses were able to explain beneficence, autonomy, nonmaleficence, and justice in various ways. Nurses demonstrated how these principles relate to pain management. All the nurses had a minimum qualification of diploma in general nursing, possibly depicting the role of higher education on knowledge of nursing ethics. Previous studies conducted proved that nurses with higher degrees in nursing had good understanding of ethical principles(30,31). Contrary findings were revealed in Ghana as they found that health professionals including nurses had low knowledge of bio-ethical principles (43). Similarly, an Ethiopian study found that nurses had inadequate knowledge of patient autonomy (31). It could be deduced that incorporating ethical principles in surgical nursing curriculum could enhance the provision of ethically acceptable nursing care.

Nurses helped patients who could not afford food and medications with money. They said that such acts were benevolent to patients which is supported by previous findings which noted that beneficence encompasses acts of charity and mercy (44). The inability of some patients to afford certain things while on admission identified in the study could be a reflection of the poor socioeconomic status of the region (45,46)which makes access to quality healthcare difficult. Even though the National Health Insurance Scheme (NHIS) is available, some medications and treatment are not covered and thus, patients have to pay for such treatment.

The nurses also made efforts to ensure patients' comfort as part of their nursing roles. Nurses spent extra work time for their patients' wellbeing. other studies have explained that beneficence is doing good and sacrificing for patients (9,7). Policies for compensating nurses who provide extra support for patients may motivate other staff. It was also found that nurses sacrificed their energy and time to care for overwhelming high numbers of patients at the expense of their own health. They indicated that it was a form of doing good to patients. Nurses being overburdened with work could lead to a state of moral distress once they may be unable

to accomplish all their roles despite their knowledge of expected duties as indicated in related studies (47).

Nurses in this current study said that they provided treatment options to patients and allowed them to make decisions. This finding is consistent with the assertion that autonomy is enabling patients to make decisions on health care interventions they want to receive (17). Furthermore, nurses in the current study said that the subjective nature of pain was best treated when autonomy of patients was upheld which supports an earlier finding that pain experience is subjective and nurses need to give patients individualised attention (21). If patients' autonomy is viewed as a right, then it can be deduced that nurses will respect patients' views. This is because it will place obligation on nurses to appreciate patients' autonomous decisions. Quality nursing care will therefore, be enhanced(48) since patients will feel valued.

The study also established that nurses empower patients through education on their disease conditions and making treatment options available. This finding supports an earlier study which showed that patients' empowerment began when professionals recognised that patients were in control of their conditions and were able to make informed decisions after educating them (22). This may imply that patient education not only provides knowledge, but also empower patients to take part in their care which can reduce morbidity as it was found that patients with hypertension had improved healthy lifestyle behaviours and decreased blood pressure after they were educated (49).

The nurses also said that they were able to make decisions independently in the absence of prescribers. It also identified that nurse autonomy include making independent judgement and having freedom to function (14). The nurses in this study knew their decision making roles. Earlier studies indicated that nurses were aware of their decision making roles (50). Some nurses made decisions with doctors and collaborated in patients' pain management. This can be presumed that nurses' autonomy is being enhanced in patient care. It also imply that teamwork has the potential of increasing nurses' autonomy which is consistent with a previous study (48). Providing measures that enable nurses to participate in decision making and collaboration in the healthcare environment will enhance autonomy and help improve quality patient care since nurses' confidence will increase (33).

Participants explained that they were being non-discriminatory and meted fair treatment to patients. Previous studies noted that nurses were being non-discriminatory to patients (51,25,52). Nurses further said that justice is being impartial irrespective of the patient's colour, race or social status (53). It was also found that being impartial to patients could be achieved through paying attention to their needs, and respecting their rights to treatment (51). As nurses

are impartial and acknowledge patients' rights to pain treatment, nurses become generous with prescribed analgesics.

Nurses also explained that advocating for patients and defending vulnerable ones were also aspects of ensuring fair treatment of patients. Nurses who advocate for patients safeguard interests and well-being of patients (28). Previous study also found that protecting patients' rights was part of advocacy (29). A recent study also concluded that, advocacy roles played by nurses may enhance patient autonomy (54), suggesting that patients who are advocated for may end up having knowledge of their rights to treatment and therefore, will expect fair treatment from nurses.

Limitations of the study

The study made use of only surgical wards of a single health facility which could limit findings from being representative of Ghanaian nurses' views. Only registered general nurses participated in the study. Other category of nurses could have different and divergent views of the ethical principles.

Implications of the study

Considerable knowledge of bio-ethical principles by nurses has the tendency to impart holistic of postoperative management which has been a long standing problem. This must be considered since conventional approaches to pain management do not typically yield desired outcomes for patients. The moral obligation to attend to patients in pain should therefore, be intrinsic and cultivated by professional nurses through internalisation the bio-ethical principles in health through training.

Conclusion

General nurses in the study understood that nurses and patients' autonomy has the potential of enhancing better patients' pain management outcome. Nurses also appreciated the need to be fair to patients by providing them with the required analgesics based on the ethical principles of justice and beneficence. Nurses went the extra mile to assist patients in various ways to secure medications and settle hospital treatment bills due to their compassionate nature.

Hospital authorities therefore, need to continue to encourage nursing staff and provide them with some sort of incentives so that nurses feel movitated and acknowledged.

Acknowledgement

Authors express sincere thanks to the management and staff of the hospital in which the study was carried. Their willingness to accommodate us for the study is highly appreciated.

References

1. Benner P, Tanner C, Chesla C. Expertise in Nursing Practice. 2nd ed. Graubard A, Druck W, editors. Vol. 63, AORN Journal. Springer publishing Company; 2009. 940–941 p.
2. Petiprin A. Nursing Code of Ethics - Nursing Theory [Internet]. 2016 [cited 2017 Jun 7]. Available from: http://www.nursing-theory.org/articles/Nursing-ethics.php
3. American Nurses Association. Code of Ethics: An Overview [Internet]. 2015 [cited 2017 Jun 7]. Available from: https://learn.ana-nursingknowledge.org/products/Code-of-Ethics-An-Overview
4. Fry ST. Ethics in nursing practice: a guide to ethical decision making. 3rd ed. Melbourne: Blackwell Publishing; 2008. 1–14 p.
5. Dnp AS, Murphy M, Dip G, Care C. Medical futility in the care of non-competent terminally ill patient: Nursing perspectives and responsibilities. Aust Crit Care [Internet]. 2012; Available from: http://dx.doi.org/10.1016/j.aucc.2012.10.003
6. Beauchamp TL, Childress JF. Principles of biomedical ethics. Oxford University Press, USA; 2001.
7. Quinlan-Colwell A. Making an ethical plan for treating patients in pain. Dimens Crit Care Nurs [Internet]. 2013;33(2):91–5. Available from: http://content.wkhealth.com/linkback/openurl?sid=WKPTLP:landingpage&an=00003465-201403000-00016
8. Alzheimer Europe. Beneficence and non-maleficence [Internet]. 2009 [cited 2017 Jun 30]. p. 3075111. Available from: http://www.alzheimer-europe.org/Ethics/Definitions-and-approaches/The-four-common-bioethical-principles/Beneficence-and-non-maleficence
9. Andersson GBJ, Chapman JR, Dekutoski MB, Dettori J, Fehlings MG, Fourney DR, et

al. Do no harm: The balance of “beneficence” and “non- Maleficence.” Vol. 35, Spine. LWW; 2010.

10. Aurelio MSG. The Duties of Mercy. Prajna Vihara. 2014;15(2).

11. Pinto S, Caldeiraˉilvia, Martins JC. A systematic literature review toward the characterization of comfort. Holist Nurs Pract. 2016;30(1):14–24.

12. Yousefi H, Abedi HA, Yarmohammadian MH, Elliott D. Comfort as a basic need in hospitalized patients in Iran: a hermeneutic phenomenology study. J Adv Nurs [Internet]. 2009 Sep;65(9):1891–8. Available from: http://doi.wiley.com/10.1111/j.1365-2648.2009.05026.x

13. Twigg DE, Gelder L, Myers H. The impact of understaffed shifts on nurse-sensitive outcomes. J Adv Nurs. 2015;71(7):1564–72.

14. Tomey AM. Nursing leadership and management effects work environments. J Nurs Manag. 2009;17(1):15–25.

15. Filipova AA. Licensed nurses’ perceptions of ethical climates in skilled nursing facilities. Nurs Ethics [Internet]. 2009;16(5):574–88. Available from: http://nej.sagepub.com/cgi/doi/10.1177/0969733009106650

16. Hinkle JL, Cheever KH. Brunner & Suddarth’s textbook of medical-surgical nursing [Internet]. 13th ed. Wolters Kluwer Health/Lippincott Williams & Wilkins,; 2014. 2244 p. Available from: https://books.google.com.pe/books?id=3u6AMAEACAAJ&dq=brunner+and+suddarth+13th+edition&hl=es&sa=X&ved=0ahUKEwjk167X-NraAhUqw1kKHTd4Bd4Q6AEIJjAA

17. Entwistle VA, Carter SM, Cribb A, McCaffery K. Supporting patient autonomy: The importance of clinician-patient relationships. J Gen Intern Med [Internet]. 2010 Jul 6 [cited 2018 May 5];25(7):741–5. Available from: http://link.springer.com/10.1007/s11606-010-1292-2

18. Berman A, Snyder SJ, Frandsen G. Fundamentals of nursing: concepts, process, and practice. Pearson Education; 2016.

19. Dreyer A, Førde R, Nortvedt P. Ethical decision-making in nursing homes: Influence of organizational factors. Nurs Ethics [Internet]. 2011 Jul 14;18(4):514–25. Available from: http://nej.sagepub.com/cgi/doi/10.1177/0969733011403553

20. McCaffery M. Nursing practice theories related to cognition, bodily pain, and man-environment interactions. University of California Print. Office; 1968.

21. Aziato L, Adejumo O. An Ethnographic Exploration of Postoperative Pain

Experiences Among Ghanaian Surgical Patients. J Transcult Nurs [Internet]. 2015;26(3):301–7. Available from: http://tcn.sagepub.com/cgi/doi/10.1177/1043659614526246

22. Anderson RM, Funnell MM. Patient empowerment: Myths and misconceptions. Patient Educ Couns [Internet]. 2010;79(3):277–82. Available from: http://dx.doi.org/10.1016/j.pec.2009.07.025

23. Deber RB, Kraetschmer N, Urowitz S, Sharpe N. Do people want to be autonomous patients? Preferred roles in treatment decision-making in several patient populations. Heal Expect. 2007;10(3):248–58.

24. Sandman L, Granger BB, Ekman I, Munthe C. Adherence, shared decision-making and patient autonomy. Med Heal Care Philos [Internet]. 2012 May 17 [cited 2018 May 5];15(2):115–27. Available from: http://link.springer.com/10.1007/s11019-011-9336-x

25. Feinsod F, Wagner C. The Ethical Principle of Justice: The Purveyor of Equality [Internet]. Annals of Long-term Care. 2008 [cited 2018 May 6]. Available from: https://www.managedhealthcareconnect.com/article/8210

26. Douglas MK, Pierce JU, Rosenkoetter M, Pacquiao D, Callister LC, Hattar-Pollara M, et al. Standards of Practice for Culturally Competent Nursing Care: 2011 Update. J Transcult Nurs. 2011;22(4):317–33.

27. Herr K, Coyne PJ, McCaffery M, Manworren R, Merkel S. Pain Assessment in the Patient Unable to Self-Report: Position Statement with Clinical Practice Recommendations. Pain Manag Nurs [Internet]. 2011;12(4):230–50. Available from: http://dx.doi.org/10.1016/j.pmn.2011.10.002

28. Choi SP pin, Cheung K, Pang SM che. A field study of the role of nurses in advocating for safe practice in hospitals. J Adv Nurs. 2014;70(7):1584–93.

29. Davoodvand S, Abbaszadeh A, Ahmadi F. Patient advocacy from the clinical nurses' viewpoint: A qualitative study. J Med Ethics Hist Med. 2016;9:1–8.

30. Osingada CP, Nalwadda G, Ngabirano T, Wakida J, Sewankambo N, Nakanjako D. Nurses' knowledge in ethics and their perceptions regarding continuing ethics education: A cross-sectional survey among nurses at three referral hospitals in Uganda. BMC Res Notes [Internet]. 2015;8(1):319. Available from: http://www.pubmedcentral.nih.gov/articlerender.fcgi?artid=4517354&tool=pmcentrez&rendertype=abstract

31. Fantahun A, Demessie A, Gebrekirstos K, Zemene A, Yetayeh G. A cross sectional study on factors influencing professionalism in nursing among nurses in. BMC Nurs.

2014;1–7.

32. Iranmanesh S, Razban F, Ghazanfari Z, Nejad AT. Nurses ’ Professional Autonomy and Attitudes Toward Caring for Dying Patients in South-East Iran. Int J Palliat Nurs. 2013;20(6):294–300.
33. Ulrich CM, Zhou QP, Hanlon A, Danis M, Grady C. The impact of ethics and work-related factors on nurse practitioners’ and physician assistants’ views on quality of primary healthcare in the United States. Appl Nurs Res [Internet]. 2014;27(3):152–6. Available from: http://dx.doi.org/10.1016/j.apnr.2014.01.001
34. Wood MJ, Ross-Kerr JC. Basic Steps in Planning Nursing Research: From Question to Proposal. 7th Ed. The American Journal of Nursing. Boston: Jones and Bartlett Publishers; 2011. 521 p.
35. Wood MJ, Ross-Kerr J. Basic steps in planning nursing research: From question to proposal. Jones & Bartlett Publishers; 2010.
36. Colorafi KJ, Evans B. Qualitative Descriptive Methods in Health Science Research. Heal Environ Res Des J. 2016;9(4):16–25.
37. Vaismoradi M, Turunen H, Bondas T. Content analysis and thematic analysis: Implications for conducting a qualitative descriptive study. Nurs Heal Sci. 2013;15(3):398–405.
38. Erlingsson C, Brysiewicz P. A hands-on guide to doing content analysis. African J Emerg Med. 2017;7(3):93–9.
39. Anney VN. Ensuring the quality of the findings of qualitative research: looking at trustworthiness criteria. J Emerg Trends Educ Res Policy Stud. 2014;5(2):272–81.
40. Polit DF, Beck CT. Essentials of Nursing Research: Appraising Evidence for Nursing Practice. 7th Ed. Philadelphia: Wolters Kluwer Health | Lippincott Williams & Wilkins.; 2010.
41. Bhattacherjee A. Social Science Research: Principles, Methods, and Practices. 2nd Ed. Florida: scholar commons, university of south Floride; 2012.
42. Lincoln YG, Guba E. E. 1985. Naturalistic Inquiry. London, Sage Publ Context Evid from Distrib Teams” Inf Syst Res. 1985;16(1):9–27.
43. Opoku, J.K., Addai-Mensah P. an Assessment of Perceptions and Knowledge of Health Care Ethical Principles Among Health Care Practitioners in the Kumasi Metropolis . Vol. 2. Kwame Nkrumah University of Science and Technology (KNUST) -Kumasi, Ghana.; 2014.
44. Aurelio MSG. The duties of mercy. Prajna Vihara. 2014;16(42):89–104.

45. Cooke E, Hague S, McKay A. The Ghana Poverty and Inequality Report-2016 [Internet]. Unicef. Accra; 2016. Available from: https://www.unicef.org/ghana/Ghana_Poverty_and_Inequality_Analysis_FINAL_Match_2016(1).pdf
46. Ghana Statistical Service. 2010 Population and Housing Census [Internet]. Accra; 2010. Available from: http://dx.plos.org/10.1371/journal.pone.0104053
47. Schluter, J., Winch, S., Holzhauser, K. & Henderson A, Fry ST. Nurses' moral sensitivity and hosptital ethical climate: A literature review. Nurs Ethics. 2008;15(3):304–21.
48. Traynor M, Boland M, Buus N. Professional autonomy in 21st century healthcare: Nurses' accounts of clinical decision-making. Soc Sci Med [Internet]. 2010 [cited 2017 Jan 6];71(8):1506–12. Available from: http://dx.doi.org/10.1016/j.socscimed.2010.07.029
49. Hacihasanoğlu R, Gözüm S. The effect of patient education and home monitoring on medication compliance, hypertension management, healthy lifestyle behaviours and BMI in a primary health care setting. J Clin Nurs. 2011;20(5–6):692–705.
50. Gallagher A, Bousso RS, McCarthy J, Kohlen H, Andrews T, Paganini MC, et al. Negotiated reorienting: A grounded theory of nurses' end-of-life decision-making in the intensive care unit. Int J Nurs Stud [Internet]. 2015 [cited 2016 Nov 14];52(4):794–803. Available from: http://dx.doi.org/10.1016/j.ijnurstu.2014.12.003
51. Alzheimer Europe. The four common bioethical principles - Alzheimer Europe [Internet]. Alzheimer Europe. 2010 [cited 2018 May 6]. p. 11–2. Available from: http://www.alzheimer-europe.org/Ethics/Definitions-and-approaches/The-four-common-bioethical-principles
52. V M Aziz, Rugiyya Saeed, Rim Roufael. The four basic ethical principles in palliative and end of life care in Old Age Psychiatry [Internet]. 2018 [cited 2018 May 6]. Available from: https://www.rcpsych.ac.uk/workinpsychiatry/faculties/oldagepsychiatry/newsletters/enewsletterjanuary2018/principlesinpalliativecare.aspx
53. Aiken LH, Sloane D, Griffiths P, Rafferty AM, Bruyneel L, McHugh M, et al. Nursing skill mix in European hospitals: Cross-sectional study of the association with mortality, patient ratings, and quality of care. BMJ Qual Saf. 2017;26(7):559–68.
54. Dadzie G, Aziato L, Graft Aikins A de. Patient Characteristics that Influence the Advocacy Role of Nurses in Ghana: A Qualitative Study. J Nurs Patient Care

[Internet]. 2018;03(01):1–5. Available from: https://www.scitechnol.com/peer-review/patient-characteristics-that-influence-the-advocacy-role-of-nurses-in-ghana-a-qualitative-study-65Ch.php?article_id=7458
