# Supplementary material for: Exploring Ghanaian nurses knowledge and application of bio-ethical principles in postoperative pain management
Source: PLoS One. 2022 Oct 19;17(10):e0276422. doi: 10.1371/journal.pone.0276422 (PMC9581380; doi:10.1371/journal.pone.0276422)
Supplement: Attachment — Submitted filename: Comments.docx [file pone.0276422.s003.docx]

The author has now related the ethical principles to pain management which is convincing the reader. Following grammatical errors need to be corrected.

**Demographics:** Page 8 - there is no need to mention the number in the bracket as it is the repetition of the word.

‘There were fourteen (14) participants including three (3) males and eleven (11) females’

Spelling error - Participants

Page 10: There is a repetition of word- **give**
