# Supplementary material for: Exploring Ghanaian nurses knowledge and application of bio-ethical principles in postoperative pain management
Source: PLoS One. 2022 Oct 19;17(10):e0276422. doi: 10.1371/journal.pone.0276422 (PMC9581380; doi:10.1371/journal.pone.0276422)
Supplement: Attachment — Submitted filename: Response to Reviewers.docx [file pone.0276422.s005.docx]

Moses Banoya Tia (corresponding author)

Methodist University College Ghana

Wenchi, Ghana.

Dear Sir,

**Response to reviewers**

I humbly submit to you the revisions required according to the reviewers. It is presented in a tabular form below.

| **Reviewer # 3** |  |
| --- | --- |
| Comment | Response |
| 1. What are the aims of objectives of this study? They should be clearly delineated rather than suggested. | The aim/objective of the study is stated in the abstract. |
| 2. What percentage of the nursing staff was included? | This has been addressed by stating the total number of nursing staff. On page 7 |
| 3. How was the sample size determined? Was it until saturation was achieved? | This has been addressed on page 7 as “interviews were conducted until the thirteenth participant when data saturation was reached. One more participant was interviewed to ensure that saturation was actually reached. Therefore, a total of 14 participants were interviewed.” |
| **Reviewer # 4** |  |
| Comment | Response |
| 1. Page 8 - there is no need to mention the number in the bracket as it is the repetition of the word | The numbers in bracket has been removed. |
| 2. ‘There were fourteen (14) participants including three (3) males and eleven (11) females’ Spelling error - Participants | The spelling error has been addressed. |
| 3. Page 10: There is a repetition of word- give | The repeated word has been deleted. |

Thank you.
