# Supplementary material for: Exploring Ghanaian nurses knowledge and application of bio-ethical principles in postoperative pain management
Source: PLoS One. 2022 Oct 19;17(10):e0276422. doi: 10.1371/journal.pone.0276422 (PMC9581380; doi:10.1371/journal.pone.0276422)
Supplement: Attachment — Submitted filename: Response to reviewers.docx [file pone.0276422.s006.docx]

Moses Banoya Tia (corresponding author)

Methodist University College Ghana

Wenchi, Ghana.

Dear Sir,

**Response to reviewers**

I humbly submit to you the revisions required according to the reviewers. It is presented in a tabular form below.

| Comment | Response |
| --- | --- |
| **Editor comment** |  |
| 1) defined objectives or research questions | The objectives of the research is stated in the abstract as “Therefore, the study objectives were to: explore nurses’ understanding of the bio-ethical principles in postoperative pain management; and explore how nurses apply bioethical principles in postoperative pain management.” |
| 2) description of the sampling strategy/sample size determination, including rationale for the recruitment method, participant inclusion/exclusion criteria and the number of participants recruited | Homogenous sampling technique has been described as “It is a sampling technique in which the researcher aims at achieving homogeneity of participants. By this, the researcher seeks to obtain a sample that has similar characteristics such as same level of education and occupation.” sample size determination is stated “Interviews were conducted until the thirteenth participant when data saturation was reached. One more participant was interviewed to ensure that saturation was actually reached.”  Inclusion and exclusion criteria is stated: “Registered general nurses with a minimum of diploma certificates and one year minimum working experience were eligible to participate. Nurses on various forms of leave were excluded from the study.”  Number of participants is mentioned as “Therefore, a total of 14 participants were interviewed.” |
| 4) a discussion of potential sources of bias and limitations | These are indicated under the “limitations of the study” of the manuscript. |
| 1) presentation style corrections (one example: methods-design-6th line:- "as suggested by (34)"; another example: "According to (39–41)") | These mistakes are addressed, example, line 6 under “design” is changed to … responses, and also owing to the fact that little is known about the topic under study (34). |
| Comment on references | References have been updated and errors corrected. |

Thank you.

Signed

Moses Banoya Tia
