# Supplementary material for: Exploring Ghanaian nurses knowledge and application of bio-ethical principles in postoperative pain management
Source: PLoS One. 2022 Oct 19;17(10):e0276422. doi: 10.1371/journal.pone.0276422 (PMC9581380; doi:10.1371/journal.pone.0276422)
Supplement: Attachment — Submitted filename: Reviewer comment.docx [file pone.0276422.s002.docx]

1. The manuscript is technically sound and well prepared.
2. Results are presented as per the objectives of the study
3. Manuscript is well written, however there are few errors noted which needs to be rectified. Comments are given in the manuscript.

Manuscript can be accepted for publication
